# Family History and Uterine Fibroid Development in Black and African American Women

**DOI:** 10.1001/jamanetworkopen.2024.4185

**Published:** 2024-04-03

**Authors:** Christine R. Langton, Quaker E. Harmon, Donna D. Baird

**Affiliations:** 1 Epidemiology Branch, National Institute of Environmental Health Sciences, Research Triangle Park, North Carolina

## Abstract

**Importance:**

Uterine fibroids are an understudied condition, with earlier onset in Black than White women. Prior studies of the importance of family history on fibroid development are limited by reliance on hospital-based participant selection, poorly defined measures of family history, and nonsystematic fibroid assessment.

**Objective:**

To examine whether family history is a risk factor for fibroid development using prospective ultrasonography data to identify incident fibroids and measure fibroid growth and standardized methods to ascertain family history.

**Design, Setting, and Participants:**

This prospective community cohort of Black and African American women from the Detroit, Michigan, area was conducted from January 1, 2010, to December 31, 2018, using 4 standardized ultrasonographic examinations during 5 years to detect fibroids 0.5 cm or larger in diameter and measure fibroid growth. Data analysis was performed between May 2022 and January 2024.

**Exposures:**

Maternal fibroid history data were gathered directly from participants’ mothers when possible (1425/1628 [88%]), and 2 exposure variables were created: maternal history of fibroids (diagnosed vs not diagnosed) and age at maternal fibroid diagnosis (20-29, 30-39, or ≥40 years vs not diagnosed).

**Main Outcomes and Measures:**

Fibroid incidence was assessed using multivariable Cox proportional hazards regression models; fibroid growth was calculated as change in log-volume per 18 months for fibroids matched at successive ultrasonograms.

**Results:**

A total of 1610 self-identified Black and/or African American women aged 23 to 35 years (mean [SD] age, 29.2 [3.4] years) with no prior clinical diagnosis of fibroids at enrollment were available for analysis. Of 1187 fibroid-free participants at enrollment, 442 (37%) had mothers who were diagnosed with fibroids. Compared with participants without a maternal history of fibroids, those reporting maternal history had an adjusted hazard ratio (AHR) of 1.21 (95% CI, 0.96-1.52). Risk was strongest in those whose mothers were diagnosed at a younger age (20-29 years: AHR, 1.56; 95% CI, 1.11-2.21; 30-39 years: AHR, 1.03; 95% CI, 0.71-1.49; ≥40 years: AHR, 1.11; 95% CI, 0.81-1.52; *P* = .053 for trend). Fibroid growth rates were higher when mothers were diagnosed with fibroids vs not diagnosed (8.0% increased growth; 95% CI, −1.2% to 18.0%).

**Conclusions and Relevance:**

In this prospective cohort study, results supported maternal history of fibroids as a risk factor for incident fibroids, especially when mothers were diagnosed at a younger age. Maternal history was also associated with increased fibroid growth. Asking patients about their family history of fibroids could encourage patient self-advocacy and inform care.

## Key Points

**Question:**

Is a family history of uterine fibroids among Black and African American women associated with increased risk in daughters?

**Findings:**

In this cohort study of 1610 Black and African American women, a maternal history of fibroids was associated with an increased risk of ultrasonography-identified incident fibroids, with a stronger association observed among those whose mothers were diagnosed at a younger age. Participants with a maternal history of fibroids also experienced an increased rate of fibroid growth.

**Meaning:**

These findings suggest that, when patients present to clinical care with symptoms that may be attributable to fibroids, collection of maternal fibroid history data could provide information for enhanced patient care.

## Teaser

This cohort study of Black and African American women assesses whether a family history of fibroids is associated with fibroid development.

## Introduction

Uterine fibroids, also known as leiomyomas, are benign tumors of the uterine muscle that develop in more than 70% of women of reproductive age.^1^ Symptomatic fibroids can result in substantial morbidity^2^ and are the leading indication for hysterectomy in the US.^3^ African American women experience fibroid onset an estimated 10 years earlier than US White women^4^ and have a disproportionate health burden from fibroids.^5^

Having a first-degree relative, primarily a mother or sister, with a history of uterine fibroids is thought to be a risk factor for fibroid development.^2,6^ Genetic associations with fibroids have been examined in familial aggregation, twin, and genome-wide association studies; most, but not all, indicate that inherited genetic factors have some influence.^7,8^ However, social and environmental factors are also associated with fibroid risk and can be shared within families; thus, it is difficult to disentangle the contribution of shared environment vs genetics among family members.^6,9^ Although few in number, prior epidemiologic studies have usually reported an increased risk if a family member has the condition.^10,11,12,13,14,15,16,17,18^ However, these studies were limited by cross-sectional methods, reliance on hospital-based participants, or surgical diagnosis of fibroids. Moreover, interpretation is challenging because the measure of family history in most studies was poorly defined. Furthermore, no studies have examined the association of family history with fibroid growth, which may be important for developing clinical care plans for patients with diagnosed fibroids.^19,20^

Our objective was to assess the association of a family history of fibroids with fibroid development among Black and African American women using prospective ultrasonographic examinations to measure fibroid incidence and growth and systematic ascertainment of family history. We hypothesized that participants’ risk of uterine fibroids may be higher if family members were diagnosed at an early age, analogous to increased risk of certain cancers when family members have early onset of the cancer.^21,22^

## Methods

The Study of Environment, Lifestyle & Fibroids (SELF), a prospective study of uterine fibroid incidence and growth among a cohort of premenopausal women with no prior clinical diagnosis of fibroids, has been described previously.^23,24^ Briefly, individuals were recruited from January 1, 2010, to December 31, 2012, in the Detroit, Michigan, area with collaboration from Henry Ford Health. SELF enrollment was limited to women who self-identified as Black or African American among a list of racial and ethnic categories. Enrolled participants (n = 1693), aged 23 to 35 years at baseline ultrasonography, were prospectively followed up for fibroid development with standardized ultrasonographic examinations at approximately 20-month intervals during 5 years for a total of 4 visits. Follow-up was concluded by December 31, 2018. Retention in the study was high, with 1543 participants (91%) attending the final visit. SELF was approved by the institutional review boards of Henry Ford Health and the National Institute of Environmental Health Sciences. All participants provided written informed consent. We followed the Strengthening the Reporting of Observational Studies in Epidemiology (STROBE) reporting guidelines for cohort studies.^25^

### Family History Assessment

We relied on maternal history of fibroids that was assessed on 2 questionnaires. On the preenrollment questionnaire, participants were asked, “Has your biological mother been diagnosed with fibroids?” with response options of yes, no, or don’t know. At the time of enrollment, participants were asked to complete 1 of 2 versions of the early-life questionnaire that included maternal information. Participants who reported being able to speak with their mother were given a version designed as an interview so the questions could be answered by participants’ mothers. Remaining participants were given a version that simply listed the questions, and they were instructed to get help from relatives and family friends. The questionnaires ascertained whether a participant’s mother was ever told by a physician or other health care professional that she had fibroids in her uterus, with response options of yes, no, or don’t know. For yes responses, the age of diagnosis was captured via 8 response categories (20-24, 25-29, 30-34, 35-39, 40-44, 45-49, or ≥50 years or don’t know).

The preenrollment questionnaire was completed by all participants and the early-life questionnaire by 1628 participants (96%), of whom 1425 (88%) got answers directly from their mothers. We created 2 exposure variables: maternal history of fibroids (diagnosed vs not diagnosed) populated by data from the early-life (1495 [97%]) and preenrollment (52 [3%]) questionnaires and age at maternal fibroid diagnosis (20-29, 30-39, or ≥40 years vs not diagnosed).

### Fibroid Assessment

Methods for the detection and measurement of fibroids in SELF have been described previously.^20,23^ Briefly, transvaginal ultrasonography was conducted by experienced sonographers using 2-dimensional imaging. Sonographers were trained on the standardized protocol to detect, measure, and document fibroids 0.5 cm or larger in diameter. All fibroids up to the 6 largest fibroids per participant were measured in 3 perpendicular planes at 3 separate passes through the uterus, and these measures were used to calculate a fibroid volume based on the ellipsoid formula. A mean of the 3 separate volume calculations was used in analysis. Video and still images were archived, and an 8% sample from each sonographer per month, oversampled for fibroid cases, was reviewed by the lead sonographer, who subsequently communicated with each sonographer to maintain consistency.

### Covariates

At enrollment, participants reported their age of menarche and the highest year or level of school completed by their mother or primary caregiver when the participants were approximately 10 years old. The mother’s age at time of participant’s enrollment into the cohort was derived from mother’s date of birth on the participant’s birth certificate (1188 [74%]), early-life questionnaire (381 [24%]), or preenrollment questionnaire (29 [2%]). Time-varying factors were assessed at each visit: use of injectable depot medroxyprogesterone acetate (DMPA), pregnancy history, cigarette use, annual household income, and body mass index (BMI; calculated as measured weight in kilograms divided by measured height in meters squared). Data on DMPA and births were used to derive years since last DMPA use and last birth, respectively.

### Analytical Cohorts

**Figure 1.**
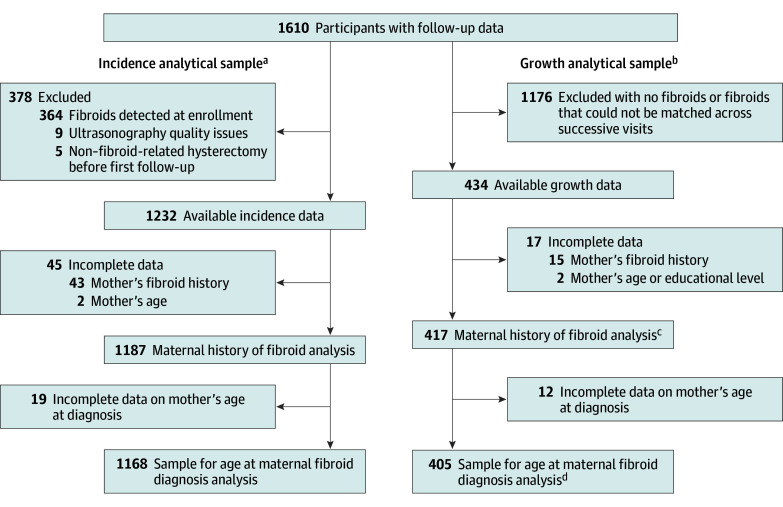
Flowchart of Participant Selection in the Study of Environment, Lifestyle & Fibroids, 2010-2018 Of the 1610 participants at enrollment with follow-up data available, a total of 1187 were included in the incidence analytical sample and 417 were included in the growth analytical sample, with 1168 and 405 participants available for the age at maternal fibroid diagnosis analysis, respectively. ^a^Participants free of fibroids at enrollment with complete data. ^b^Participants with fibroids detected at enrollment or during follow-up with complete data. ^c^Includes 1314 fibroid growth intervals. ^d^Includes 1272 fibroid growth intervals.

A total of 1610 participants (95%) had 1 or more follow-up visits and were available for the incidence and/or growth analyses (Figure 1). Although participants had not been previously diagnosed, 364 (23%) had fibroids at their baseline ultrasonography. For the incidence analysis, these participants were excluded along with 5 participants who had a non–fibroid-related hysterectomy before their first follow-up and 9 with ultrasonographic visualization issues, resulting in 1232 participants. After excluding participants missing maternal fibroid history (n = 43) and important covariates (n = 2), our final incidence analytical data set comprised 1187 participants. Participants in the growth sample included those with fibroids detected at enrollment (n = 300) or during follow-up (n = 117) who had fibroids that could be matched across successive visits using archived images and fibroid location^20^ and who were not missing maternal fibroid history (n = 15) or covariate (n = 2) data, resulting in 417 participants.

### Statistical Analysis

To examine the associations between maternal fibroid history and fibroid incidence, we used Cox proportional hazards regression with participant age as the time scale to estimate adjusted hazard ratios (AHRs) and 95% CIs. Maternal history of fibroids was modeled as a binary variable, and age at maternal diagnosis was modeled using 4 categories (not diagnosed and diagnosed at 20-29, 30-39, or ≥40 years of age). Linear trends were assessed by modeling the age at maternal diagnosis variable as ordinal with categories in order of hypothesized risk increase (not diagnosed and diagnosed at ≥40, 30-39, or 20-29 years of age). Incident fibroid cases were participants who had fibroid(s) detected at a follow-up visit. Participants contributed follow-up time until they had an incident fibroid, non–fibroid-related hysterectomy, loss to follow-up, or the final study visit, whichever came first.

To control for confounding, we created 3 multivariable models with potential confounders identified from previous literature^8,14,17^ and associations with fibroids in the cohort.^24^ All models were adjusted for participant age by using age as the time scale. First, we ran a model only adjusted for age. Second, we adjusted for maternal factors that may have influenced likelihood of a clinical diagnosis (mother’s age at time of participant’s enrollment into the study [continuous] and highest educational attainment of the mother when participant was approximately 10 years old [high school or General Educational Development or less vs some college or technical training after high school or college degree]).^5^ Third, we additionally adjusted for participant factors of age at menarche (≤10, 11, 12, 13, or ≥14 years) and time-varying annual household income (<$20 000 or≥$20 000), current smoker status (dichotomous), BMI (<25.0, 25.0 to <30.0, 30.0 to <35.0, 35.0 to <40.0, or ≥40), parity (0, 1-2, or ≥3 births), years since last birth (<3 vs ≥3 years or no births), and years since last DMPA use (<2 vs ≥2 years including never). Due to low missingness (0%-3.5%), we conducted complete-case analyses. We plotted and inspected Schoenfeld residuals from our fully adjusted models to test the proportionality of hazards assumption^26^; a violation of assumptions was not suggested.

For fibroid growth, we conducted a by-fibroid analysis, with fibroid growth calculated as the change in the natural logarithm of each fibroid volume and scaled to a growth rate per 18 months (median [IQR] interval length between visits, 19 [18-20] months). Associations between maternal fibroid history and fibroid growth were analyzed using linear mixed models,^19^ and details have been previously published.^20,24^ The random-effects portion of the models accounted for correlation among fibroids from the same participant, correlation over time for the same fibroid, and greater variability among volume measures for small compared with large fibroids.^20^ For increased interpretability, the logarithmic growth rate scale was back-transformed ([exp(β) − 1] × 100) to estimate the percentage difference in volume change per 18 months for those with vs without maternal history of fibroids. All fibroid growth models were adjusted for fibroid volume and number of fibroids in the uterus,^20^ as well as for covariates considered in the 3 models as described for the incidence analysis.

We conducted several sensitivity analyses for both incidence and growth findings. First, we restricted the data sets to only those participants whose mothers provided their own fibroid history to account for potential bias related to the source of maternal fibroid data. Second, we restricted the analytical data sets to participants whose mothers were 50 years or older to account for mothers younger than 50 years having less chance of being diagnosed at the older age categories. Third, participants with missing data for mother’s age at fibroid diagnosis were assigned to the youngest (20-29 years) and oldest (≥40 years) diagnosis categories in separate analyses. Fourth, to assess potential selection bias, we excluded participants who indicated family history as 1 of the top 3 reasons for joining the study. Fifth, for the growth analysis, we excluded fibroids that had residuals for growth more than 3 SDs from the mean to exclude outlier effects.^19,20^

All statistical analyses were conducted with SAS software, version 9.4 (SAS Institute Inc). Based on American Statistical Association guidance,^27^ we interpret our results based on magnitude of the point estimates and width of the CI, not on binary statistical testing. Statistical significance was set at a 2-tailed *P* < .05 for trend analysis.

## Results

A total of 1610 self-identified Black and/or African American women aged 23 to 35 years (mean [SD] age, 29.2 [3.4] years) with no prior clinical diagnosis of fibroids at enrollment were included in the analyses. Maternal and participant characteristics for the incidence (n = 1187) and growth samples (n = 417) are presented in Table 1. Characteristics by age at maternal diagnosis for the incidence and growth samples are presented in eTables 1 and 2 in Supplement 1, respectively. In the incidence sample, 37% of participants (442 of 1187) had mothers with fibroids, of whom 24% (100 of the 423 with data on mother's age at fibroid diagnosis) were diagnosed between 20 and 29 years of age, whereas in the growth sample, 44% (182 of 417) had mothers with fibroids, of whom 27% (46 of the 170 with data available) were diagnosed between 20 and 29 years of age. Overall, educational attainment was higher for both mothers and participants when the mother had a fibroid diagnosis compared with participants whose mother was not diagnosed as having fibroids. Participants whose mother had fibroids had higher annual household incomes and were less likely to be current smokers compared with those with undiagnosed mothers. Parity, alcohol use, BMI, and use of DMPA were similar irrespective of the mother’s fibroid diagnosis, and participant characteristics were mostly similar among the 3 groups of maternal age at fibroid diagnosis.

**Table 1.** Enrollment Characteristics by Maternal Uterine Fibroid Diagnosis in the Study of Environment, Lifestyle & Fibroids, 2010-2012^a^

Characteristic	Incidence analytical sample (n = 1187)		Growth analytical sample (n = 417)^b^	
	No maternal diagnosis	Maternal diagnosis	No maternal diagnosis	Maternal diagnosis
Exposure group totals	745 (63)	442 (37)	235 (56)	182 (44)
Mothers				
Age at participant enrollment, median (IQR), y	53 (48-57)	53 (49-58)	54 (51-59)	55 (52-58)
Educational attainment^c^				
High school or GED or less	386 (52)	162 (37)	120 (51)	61 (34)
Some college or technical training after high school or college degree	286 (38)	226 (51)	87 (37)	86 (47)
Bachelor’s, master’s, or doctoral degree	73 (10)	54 (12)	28 (12)	35 (19)
Age at fibroid diagnosis, y^d^				
20-29	NA	100 (24)	NA	46 (27)
30-39	NA	143 (34)	NA	59 (35)
≥40	NA	180 (43)	NA	65 (38)
Participants				
Age at enrollment, median (IQR), y	29 (26-31)	29 (26-32)	30 (28-33)	30 (28-33)
Age range at enrollment, y				
23-25	203 (27)	103 (23)	28 (12)	23 (13)
26-28	181 (24)	124 (28)	53 (23)	48 (26)
29-31	210 (28)	100 (23)	78 (33)	52 (29)
32-35	151 (20)	115 (26)	76 (32)	59 (32)
Age at menarche, mean (SD), y	12.0 (1.8)	12.0 (1.8)	11.9 (1.7)	11.7 (1.8)
Age at menarche, y				
≤10	129 (17)	77 (17)	44 (19)	48 (26)
11	148 (20)	94 (21)	49 (21)	40 (22)
12	209 (28)	123 (28)	66 (28)	38 (21)
13	127 (17)	68 (15)	46 (20)	25 (14)
≥14	132 (18)	80 (18)	30 (13)	31 (17)
Educational attainment				
High school or GED or less	206 (28)	64 (14)	50 (21)	18 (10)
Some college or technical training after high school or college degree	393 (53)	219 (50)	118 (50)	81 (45)
Bachelor’s, master’s, or doctoral degree	146 (20)	159 (36)	67 (29)	83 (46)
Annual household income, $				
<20 000	396 (53)	150 (34)	109 (46)	53 (29)
20 000-50 000	257 (35)	203 (46)	85 (36)	73 (40)
>50 000	92 (12)	89 (20)	41 (17)	56 (31)
Parity				
0 Births	252 (34)	183 (41)	105 (45)	97 (53)
1-2 Births	343 (46)	186 (42)	98 (42)	73 (40)
≥3 Births	150 (20)	73 (17)	32 (14)	12 (7)
Time since last birth				
Within 3 y	199 (27)	99 (22)	33 (14)	29 (16)
≥3 y Ago or no births	546 (73)	343 (78)	202 (86)	153 (84)
Smoking status				
Non or former	582 (78)	379 (86)	181 (77)	165 (91)
Current	163 (22)	63 (14)	54 (23)	17 (9)
Alcohol use^e^				
0 to <10 Drinks per year	228 (31)	127 (29)	71 (30)	48 (26)
Moderate	361 (48)	241 (55)	121 (51)	102 (56)
Heavy	156 (21)	74 (17)	43 (18)	32 (18)
BMI				
<25.0	144 (19)	93 (21)	40 (17)	38 (21)
25.0 to <30.0	159 (21)	90 (20)	49 (21)	34 (19)
30.0 to <35.0	133 (18)	88 (20)	48 (20)	44 (24)
35.0 to <40.0	134 (18)	70 (16)	40 (17)	33 (18)
≥40	175 (23)	101 (23)	58 (25)	33 (18)
DMPA use				
Never used	376 (50)	262 (59)	158 (67)	128 (70)
<2 y since last use	105 (14)	44 (10)	13 (6)	9 (5)
≥2 y since last use	264 (35)	136 (31)	64 (27)	45 (25)

Abbreviations: BMI, body mass index (calculated as measured weight in kilograms divided by measured height in meters squared); DMPA, depot medroxyprogesterone acetate; GED, General Educational Development; NA, not applicable.

^a^ Data are presented as number (percentage) of participants unless otherwise indicated.

^b^ Participants in the growth sample are those with fibroids detected at enrollment (n = 300) or during follow-up (n = 117) whose fibroids could be matched across successive visits.

^c^ Mother’s educational attainment at approximately 10 years of age of the participant.

^d^ Mother’s age at fibroid diagnosis missing for 19 participants in the incidence sample and 12 participants in the growth sample.

^e^ Moderate indicates 1 to 5 drinks on days when alcohol is consumed or 4 drinks or more on an occasion no more than once per month; heavy indicates 6 drinks or more on days when alcohol is consumed or 4 drinks or more on an occasion at least twice per month.

During 5121 person-years of follow-up, 285 of the 1187 fibroid-free participants at enrollment (24%) developed incident fibroids (Table 2). In analyses adjusted for age, maternal history of fibroids was associated with a modestly higher risk of incident fibroids compared with no history (model 1: AHR, 1.26; 95% CI, 1.00-1.58). Adjustment for maternal factors did not materially affect risk estimates (model 2), but the association was slightly attenuated when additionally adjusted for participant factors (model 3: AHR, 1.21; 95% CI, 0.96-1.52). When age at maternal fibroid diagnosis was considered, risk was strongest for participants whose mothers had fibroids diagnosed at earlier ages (model 1, 20-29 years: AHR, 1.63; 95% CI, 1.15-2.32; 30-39 years: AHR, 1.07; 95% CI, 0.75-1.53; ≥40 years: AHR, 1.15; 95% CI, 0.83-1.58; *P* = .03 for trend). These estimates were also slightly attenuated when adjusted for maternal and participant characteristics (model 3, 20-29 years: AHR, 1.56; 95% CI, 1.11-2.21; 30-39 years: AHR, 1.03; 95% CI, 0.71-1.49; ≥40 years: AHR 1.11; 95% CI, 0.81-1.52; *P* = .053 for trend).

**Table 2.** Multivariable Associations of Maternal History of Fibroids With Fibroid Incidence in the Study of Environment, Lifestyle & Fibroids, 2010-2018

Exposure	No. of participants	No. of incident cases	No. of person-years	HR (95% CI)		
				Model 1^a^	Model 2^b^	Model 3^c^
**Maternal history of fibroids**						
Not diagnosed	745	161	3231	1 [Reference]	1 [Reference]	1 [Reference]
Diagnosed	442	124	1890	1.26 (1.00-1.58)	1.25 (1.00-1.58)	1.21 (0.96-1.52)
**Age at maternal fibroids diagnosis** ^d^						
Not diagnosed	745	161	3231	1 [Reference]	1 [Reference]	1 [Reference]
20-29 y	100	33	427	1.63 (1.15-2.32)	1.63 (1.15-2.32)	1.56 (1.11-2.21)
30-39 y	143	34	610	1.07 (0.75-1.53)	1.07 (0.75-1.53)	1.03 (0.71-1.49)
≥40 y	180	49	775	1.15 (0.83-1.58)	1.15 (0.83-1.58)	1.11 (0.81-1.52)

Abbreviation: HR, hazard ratio.

^a^ Cox proportional hazards regression model with age as the time scale and no further adjustment.

^b^ Model 1 plus adjustment for mother’s age at participant enrollment (continuous) and highest educational level of mother at 10 years of age of the participant (high school or General Educational Development or less vs some college or technical training after high school or college degree).

^c^ Model 2 plus additional adjustment for participant factors: age at menarche (≤10, 11, 13, or ≥14 vs 12 years) and time-varying factors of parity (1-2 or ≥3 vs 0 births), years since last birth (≥3 years ago including no births vs <3 years ago), years since last use of injection contraceptive (<2 vs ≥2 years including never), current smoker status (yes vs no), body mass index (calculated as measured weight in kilograms divided by measured height in meters squared) (25.0-<30.0, 30.0-<35.0, 35.0-<40.0, or ≥40.0 vs <25.0 kg/m^2^), and annual household income (<$20 000 vs ≥$20 000).

^d^ A total of 1168 participants and 277 incident cases were included in the age at maternal fibroid diagnosis models due to mother’s age at fibroid diagnosis missing for 19 participants.

**Figure 2.**
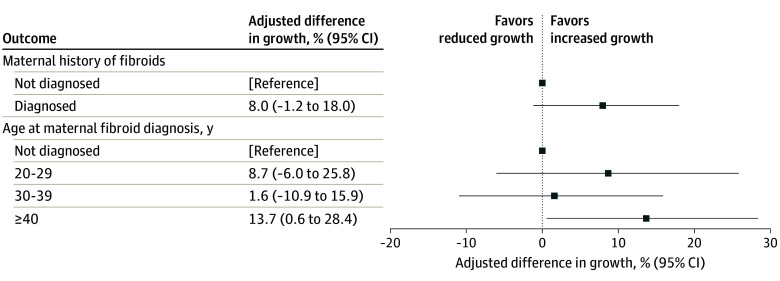
Multivariable Associations of Maternal History of Fibroids With Fibroid Growth in the Study of Environment, Lifestyle & Fibroids, 2010-2018 The figure shows an association of maternal history of fibroids with fibroid growth. Squares represent the estimated percentage difference in growth and the lines capture the 95% CI from the comparison of participants with a maternal diagnosis of uterine fibroids to participants with no maternal fibroid diagnosis. The estimates are adjusted for fibroid characteristics of fibroid volume (<0.5, 0.5-<4.2, or 4.2-<14.1 vs ≥14.1 cm^3^) and number of fibroids (ordinal: 1, 2, 3, and ≥4), maternal characteristics of mother’s age at enrollment of participant (continuous) and mother’s educational level (high school or General Educational Development or less vs some college or college degree), and participant factors: age at menarche (≤10, 11, 13, or ≥14 vs 12 years), and time-varying factors of age (continuous), body mass index (calculated as measured weight in kilograms divided by measured height in meters squared) (25.0-<30.0, 30.0-<35.0, 35.0-<40.0, or ≥40.0 vs <25.0), annual household income (<$20 000 vs ≥$20 000), current smoking (yes vs no), parity (1-2 or ≥3 vs 0 births), years since last birth (≥3 years ago including no births vs <3 years ago), and years since last use of injection contraceptive (<2 vs ≥2 years including never).

Fibroids followed for growth were generally small (median [IQR] volume, 2.2 [0.7-8.5] cm^3^; median [IQR] diameter, 1.6 [1.1-2.5] cm) and estimated mean fibroid growth was 69% volume increase per 18 months (95% CI, 61%-77%). The estimated difference in growth rate per 18 months was 8.0% (95% CI, −1.2% to 18.0%) among participants with a maternal history of fibroids compared with those without (model 3) (Figure 2; eTable 3 in Supplement 1). Percentage difference was also positive when examined by age at maternal diagnosis (model 3, 20-29 years: 8.7%; 95% CI, −6.0% to 25.8%; 30-39 years: 1.6%; 95% CI, −10.9% to 15.9%; ≥40 years: 13.7%; 95% CI, 0.6% to 28.4%), but the CIs were broad and mother’s age at diagnosis was less important.

Results from our sensitivity analyses for both incidence (eTable 4 in Supplement 1) and growth analyses (eTable 5 in Supplement 1) were mostly similar to our main findings. However, the growth estimates among the subset of participants whose mothers were 50 years or older at enrollment were strengthened, especially for those whose mothers were 40 years or older at diagnosis (model 3: percentage difference, 17.6% vs 13.7%).

## Discussion

In this community-recruited cohort of young Black and/or African American women, maternal history of fibroids was associated with an increased risk of ultrasonography-identified incident fibroids. The strongest association was observed for participants whose mothers had fibroids diagnosed at a younger age. Fibroid growth rates also tended to be increased when participants’ mothers were diagnosed with fibroids, but age at maternal diagnosis was not as important for fibroid growth.

To our knowledge, one other prospective study to date has examined family history and a fibroid outcome.^14^ In the California Teachers Study, researchers conducted an 11-year follow-up of 80 204 participants aged 25 to 80 years who reported no previous history of fibroids at baseline, recording those with a primary hospital discharge diagnosis of surgically confirmed fibroids. They observed that those with a primary surgical diagnosis of fibroids were more likely than those without to have a mother or sister with fibroids (relative risk, 1.37; 95% CI, 1.21-1.55), but age at diagnosis for family members was not available. Although these findings provide some evidence of an association between family history and fibroid outcomes, undergoing surgical treatment depends on many factors beside fibroid status, so results may be a biased estimate of an actual family history association. The remainder of prior epidemiologic research is composed of cross-sectional^11,13^ and case-control^10,12,15,16,17,18^ studies that consistently observed an association between family history and fibroid diagnosis, with estimates ranging from 20% increased risk (95% CI, 1.1-1.3) among 660 African American and/or Black participants of an urban health plan^13^ to a greater than 4-fold increased odds (OR, 4.6; 95% CI, 1.6-13.6) in a study of 193 Haitian women.^11^ In some of these studies, participant selection was predicated by hospital admission or outcome criteria that relied on surgical intervention,^15,16,17^ which raises questions about bias due to access to care and about the extent of generalizability.^28^

Overall, the definition and ascertainment methods of family history in these prior studies were unclear. For example, some simply stated family history as the exposure and provided no further details about what relatives were included as evidence for a positive family history.^10,11,12^ Others stated that the participant self-reported whether their mother, sisters, or daughters had fibroids,^13,14^ but these measures are dependent on how many of these relatives the participant had, which was not taken into account. None reported that the participant queried their family members for this information. Our study focused on the biological mother’s fibroid diagnosis, and 88% of participants were able to provide the data directly from their mother. Furthermore, this is the only study, to our knowledge, of fibroid development to examine mother’s age at fibroid diagnosis. Thus, our study extends the literature by examining maternal history of fibroids in an ultrasonography-based, prospective cohort study with well-measured family history data.

We are unable to compare our growth results because no other studies examined family history and fibroid growth. Two studies^29,30^ compared fibroid characteristics at surgery between participants with and without family history and reported no difference in fibroid size between the groups, but sample sizes were small, with approximately 30 in the family history groups.

### Clinical and Research Implications

Family history screening is an effective tool for presymptomatic risk assessment of genetic disease.^31,32^ Given the high prevalence of fibroids and the contribution of environmental and reproductive factors to fibroid development,^6,8^ family history alone is not a strong screening tool. Proactive discussion of fibroid symptoms and encouragement for patients to discuss fibroids with family members may be the most important clinical tool; resources are available that promote patient self-advocacy and provide practical information to facilitate these discussions.^33,34^ Nonetheless, information on family history of fibroids could provide insight to influence further assessment when patients with undiagnosed conditions present with symptoms common to various reproductive disorders and for determining how frequently patients with diagnosed fibroids will be monitored.^35^

Fibroids are an understudied condition,^28^ and there is still much to be elucidated concerning genetic and environmental factors and their interactions that influence development.^36,37^ For example, a recent genome-wide association study reported that age at menarche mediates the proposed causal effects of genetic variants on fibroids.^38^ Future genetic research may benefit from incorporation of data on the mother’s history of fibroids. Pregnancy cohorts that collect detailed data on mothers and offspring may also be an ideal source for future research. Ultrasonographic screening for fibroids along with self-reported lifestyle factors from both mothers and daughters would provide a rich data source to explore shared lifestyle factors, such as exercise, dietary preferences, and smoking, as well as genetic factors that are reflected in family history and likely influence fibroid development.^6,8,39^

### Limitations

This study has some limitations. Although collection of data on maternal history of fibroids directly from mothers is an improvement over prior studies, the mothers’ reports in this study were based on a clinical diagnosis. Considering barriers to timely and quality reproductive health care experienced by African American women in the US along with a generally high prevalence of undiagnosed fibroids,^2,35,40^ there is a strong likelihood that some mothers may have been categorized as undiagnosed yet truly had fibroids. In addition, family history reflects genetic as well as social and environmental factors shared between mothers and daughters. To account for potential disparities influencing prior fibroid diagnosis in the mothers, as well as lifestyle and environmental factors associated with fibroid development of the participants, we adjusted for maternal socioeconomic factors and several participant factors in our statistical models, but other factors stemming from systemic racism that could influence maternal diagnoses were not available. Although the young age and homogeneity of ethnic identity in our study population are important for identifying risk factors that contribute to earlier onset of fibroids among Black and African American women, the generalizability of our findings may be limited. Future examination of these associations with data collected until menopause and in more ethnically diverse populations is important. Finally, because risk associated with family history reflects both shared genetics and shared social and environmental factors, findings may differ in study populations for which the environmental factors that are shared across generations are different from those in our study group.

## Conclusions

Results from this large, ultrasonography-based, prospective cohort study support maternal history of fibroids as a risk factor for incident fibroids, especially if the mother is diagnosed at an early age. Fibroid growth tended to be greater among participants with maternal history of fibroids regardless of mothers’ age at diagnosis. Asking patients about their family history of fibroids could encourage patient self-advocacy and inform care.

## Notes

Supplement 1. **eTable 1.** Enrollment Characteristics of Incidence Analytical Sample by Age at Maternal Uterine Fibroid Diagnosis in the Study of Environment, Lifestyle & Fibroids, 2010-2012 **eTable 2.** Enrollment Characteristics of Growth Analytical Sample by Age at Maternal Uterine Fibroid Diagnosis in the Study of Environment, Lifestyle & Fibroids, 2010-2012 **eTable 3.** Multivariable Associations of Maternal History of Fibroids With Fibroid Growth, Study of Environment, Lifestyle & Fibroids, 2010-2018 **eTable 4.** Sensitivity Analyses for Multivariable Association of Maternal History of Fibroids With Fibroid Incidence, Study of Environment, Lifestyle & Fibroids (SELF), 2010-2018 **eTable 5.** Sensitivity Analyses for Multivariable Association of Maternal History of Fibroids With Fibroid Growth, Study of Environment, Lifestyle & Fibroids (SELF), 2010-2018

Supplement 2.
Data Sharing Statement
