# Supplementary material for: Family History and Uterine Fibroid Development in Black and African American Women
Source: JAMA Netw Open. 2024 Apr 3;7(4):e244185. doi: 10.1001/jamanetworkopen.2024.4185 (PMC10993075; doi:10.1001/jamanetworkopen.2024.4185)
Supplement: Supplement 1. — eTable 1. Enrollment Characteristics of Incidence Analytical Sample by Age at Maternal Uterine Fibroid Diagnosis in the Study of Environment, Lifestyle & Fibroids, 2010-2012 eTable 2. Enrollment Characteristics of Growth Analytical Sample by Age at Maternal Uterine Fibroid Diagnosis in the Study of Environment, Lifestyle & Fibroids, 2010-2012 eTable 3. Multivariable Associations of Maternal History of Fibroids With Fibroid Growth, Study of Environment, Lifestyle & Fibroids, 2010-2018 eTable 4. Sensitivity Analyses for Multivariable Association of Maternal History of Fibroids With Fibroid Incidence, Study of Environment, Lifestyle & Fibroids (SELF), 2010-2018 eTable 5. Sensitivity Analyses for Multivariable Association of Maternal History of Fibroids With Fibroid Growth, Study of Environment, Lifestyle & Fibroids (SELF), 2010-2018 [file jamanetwopen-e244185-s001.pdf]

Supplementary Online Content

Langton CR, Harmon QE, Baird DD. Family history and uterine fibroid development in Black and African American women. *JAMA Netw Open*. 2024;7(3):e244185. doi:10.1001/jamanetworkopen.2024.4185

eTable 1. Enrollment Characteristics of Incidence Analytical Sample by Age at Maternal Uterine Fibroid Diagnosis in the Study of Environment, Lifestyle & Fibroids, 2010-2012
eTable 2. Enrollment Characteristics of Growth Analytical Sample by Age at Maternal Uterine Fibroid Diagnosis in the Study of Environment, Lifestyle & Fibroids, 2010-2012
eTable 3. Multivariable Associations of Maternal History of Fibroids With Fibroid Growth, Study of Environment, Lifestyle & Fibroids, 2010-2018
eTable 4. Sensitivity Analyses for Multivariable Association of Maternal History of Fibroids With Fibroid Incidence, Study of Environment, Lifestyle & Fibroids (SELF), 2010-2018
eTable 5. Sensitivity Analyses for Multivariable Association of Maternal History of Fibroids With Fibroid Growth, Study of Environment, Lifestyle & Fibroids (SELF), 2010-2018

This supplementary material has been provided by the authors to give readers additional information about their work.

© 2024 Langton CR et al. *JAMA Network Open.*

eTable 1. Enrollment Characteristics of Incidence Analytical Sample by Age at Maternal Uterine Fibroid Diagnosis in the Study of Environment, Lifestyle & Fibroids, 2010-2012 (n=1168)[a,b]

Characteristic	No maternal Diagnosis	Maternal diagnosis age 20-29 y	Maternal diagnosis age 30-39 y	Maternal diagnosis age ≥40 y
Exposure group totals	745 (64)	100 (9)	143 (12)	180 (15)
**Mothers**				
Age at participant enrollment, median (IQR), y	53 (48-57)	52 (47-56)	53 (49-58)	54 (50-58)
Educational attainment[c]				
High school or GED or less	386 (52)	38 (38)	55 (38)	60 (33)
Some college or technical training after high school or college degree	286 (38)	51 (51)	72 (50)	94 (52)
Bachelor’s, master’s, or doctoral degree	73 (10)	11 (11)	16 (11)	26 (14)
**Participant**				
Age at enrollment, median (IQR), y	29 (26-31)	28 (26-31)	30 (26-32)	29 (26-32)
Age range at enrollment, y				
23-25	203 (27)	30 (30)	31 (22)	39 (22)
26-28	181 (24)	31 (31)	38 (27)	50 (28)
29-31	210 (28)	18 (18)	35 (24)	43 (24)
32-35	151 (20)	21 (21)	39 (27)	48 (27)
Age at menarche, mean (SD), y	12.0 (1.8)	12.1 (1.9)	11.9 (1.8)	12.1 (1.7)
Age at menarche, y				
≤10	129 (17)	17 (17)	27 (19)	29 (16)
11	148 (20)	21 (21)	37 (26)	32 (18)
12	209 (28)	29 (29)	33 (23)	52 (29)
13	127 (17)	16 (16)	18 (13)	33 (18)
≥14	132 (18)	17 (17)	28 (20)	34 (19)
Educational attainment				
High school or GED or less	206 (28)	14 (14)	21 (15)	25 (14)
Some college or technical training after high school or college degree	393 (53)	36 (36)	84 (59)	89 (49)
Bachelor’s, master’s, or doctoral degree	146 (20)	50 (50)	38 (27)	66 (37)
Annual household income, $				

© 2024 Langton CR et al. *JAMA Network Open.*

<20,000	396 (53)	33 (33)	47 (33)	60 (33)
20,000-50,000	257 (35)	44 (44)	69 (48)	85 (47)
>50,000	92 (12)	23 (23)	27 (19)	35 (19)
Parity				
0 births	252 (34)	48 (48)	55 (38)	78 (43)
1-2 births	343 (46)	41 (41)	64 (45)	69 (38)
≥3 births	150 (20)	11 (11)	24 (17)	33 (18)
Time since last birth				
Within 3 y	199 (27)	20 (20)	30 (21)	43 (24)
≥3 y Ago or no births	546 (73)	80 (80)	113 (79)	137 (76)
Smoking status				
Non or former	582 (78)	88 (88)	121 (85)	154 (86)
Current	163 (22)	12 (12)	22 (15)	26 (14)
Alcohol use[d]				
0-<10 Drinks per year	228 (31)	27 (27)	40 (28)	55 (31)
Moderate	361 (48)	60 (60)	76 (53)	94 (52)
Heavy	156 (21)	13 (13)	27 (19)	31 (17)
BMI				
<25.0	144 (19)	22 (22)	29 (20)	37 (21)
25.0-<30.0	159 (21)	19 (19)	28 (20)	40 (22)
30.0-<35.0	133 (18)	24 (24)	26 (18)	35 (19)
35.0-<40.0	134 (18)	17 (17)	21 (15)	30 (17)
≥ 40	175 (23)	18 (18)	39 (27)	38 (21)
DMPA use				
Never used	376 (50)	65 (65)	85 (59)	105 (58)
<2 y since last use	105 (14)	10 (10)	13 (9)	19 (11)
≥2 y since last use	264 (35)	25 (25)	45 (31)	56 (31)

Abbreviations: BMI, body mass index (calculated as measured weight in kilograms divided by measured height in meters squared); DMPA, depot medroxyprogesterone acetate; GED, General Educational Development; IQR, interquartile range; SD, standard deviation.

[a] The incidence analytical sample includes 1,187 participants, but only 1,168 are displayed here because age at mother's fibroid diagnosis was missing for 19 participants.

[b]Data are presented as number (percentage) of participants unless otherwise indicated.

[c] Mother's educational attainment at approximately 10 years of age of the participant.

© 2024 Langton CR et al. *JAMA Network Open.*

[d] Moderate indicates 1 to 5 drinks on days when alcohol is consumed or 4 drinks or more on an occasion no more than once per month; heavy indicates 6 drinks or more on days when alcohol is consumed or 4 drinks or more on an occasion at least twice per month.

© 2024 Langton CR et al. *JAMA Network Open.*

eTable 2. Enrollment Characteristics of Growth Analytical Sample by Age at Maternal Uterine Fibroid Diagnosis in the Study of Environment, Lifestyle & Fibroids, 2010-2012 (n=405)[a,b]

Characteristic	No maternal diagnosis	Maternal diagnosis age 20-29 y	Maternal diagnosis age 30-39 y	Maternal diagnosis age ≥40 y
Exposure group totals	235 (58)	46 (11)	59 (15)	65 (16)
**Mothers**				
Age at participant enrollment, median (IQR), y	54 (51-59)	54 (51-57)	56 (52-59)	55 (52-58)
Educational attainment[c]				
High school or GED or less	120 (51)	14 (30)	22 (37)	21 (32)
Some college or technical training after high school or college degree	87 (37)	24 (52)	24 (41)	33 (51)
Bachelor’s , master’s, or doctoral degree	28 (12)	8 (17)	13 (22)	11 (17)
**Participant**				
Age at enrollment, median (IQR), y	30 (28-33)	30 (28-33)	31 (28-33)	30 (27-32)
Age range at enrollment, y				
23-25	28 (12)	6 (13)	7 (12)	9 (14)
26-28	53 (23)	15 (33)	13 (22)	16 (25)
29-31	78 (33)	12 (26)	17 (29)	23 (35)
32-35	76 (32)	13 (28)	22 (37)	17 (26)
Age at menarche, mean (SD), y	11.9 (1.7)	11.4 (1.8)	11.9 (1.8)	11.8 (1.9)
Age at menarche, y				
≤10	44 (19)	14 (30)	14 (24)	15 (23)
11	49 (21)	11 (24)	14 (24)	13 (20)
12	66 (28)	11 (24)	7 (12)	15 (23)
13	46 (20)	4 (9)	11 (19)	10 (15)
≥14	30 (13)	6 (13)	13 (22)	12 (18)
Educational attainment				
High school or GED or less	50 (21)	5 (11)	4 (7)	7 (11)
Some college or technical training after high school or college degree	118 (50)	21 (46)	26 (44)	29 (45)
Bachelor’s, master’s, or doctoral degree	67 (29)	20 (43)	29 (49)	29 (45)
Annual household income, $				
<20,000	109 (46)	13 (28)	19 (32)	15 (23)

© 2024 Langton CR et al. *JAMA Network Open.*

20,000-50,000	85 (36)	19 (41)	24 (41)	28 (43)
>50,000	41 (17)	14 (30)	16 (27)	22 (34)
Parity				
0 births	105 (45)	22 (48)	30 (51)	41 (63)
1-2 births	98 (42)	21 (46)	27 (46)	20 (31)
≥3 births	32 (14)	3 (7)	2 (3)	4 (6)
Time since last birth				
Within 3 y	33 (14)	7 (15)	9 (15)	11 (17)
≥3 y ago, or no births	202 (86)	39 (85)	50 (85)	54 (83)
Smoking status				
Non or former	181 (77)	43 (93)	51 (86)	60 (92)
Current	54 (23)	3 (7)	8 (14)	5 (8)
Alcohol use[d]				
0-<10 Drinks per year	71 (30)	13 (28)	12 (20)	21 (32)
Moderate	121 (51)	23 (50)	39 (66)	31 (48)
Heavy	43 (18)	10 (22)	8 (14)	13 (20)
BMI				
<25.0	40 (17)	10 (22)	16 (27)	9 (14)
25.0-<30.0	49 (21)	3 (7)	12 (20)	16 (25)
30.0-<35.0	48 (20)	14 (30)	10 (17)	17 (26)
35.0-<40.0	40 (17)	11 (24)	12 (20)	8 (12)
≥ 40	58 (25)	8 (17)	9 (15)	15 (23)
DMPA use				
Never used	158 (67)	29 (63)	44 (75)	49 (75)
<2 y since last use	13 (6)	2 (4)	2 (3)	4 (6)
≥2 y since last use	64 (27)	15 (33)	13 (22)	12 (18)

Abbreviations: BMI, body mass index (calculated as measured weight in kilograms divided by measured height in meters squared); DMPA, depot medroxyprogesterone acetate; GED, General Educational Development; IQR, interquartile range; SD, standard deviation.

[a] Participants in the growth sample are those with fibroids detected at enrollment (n=300) or during follow-up (n=117) whose fibroids could be matched across successive visits. The growth analytical sample includes 417 participants, but 405 participants are displayed here because age at mother's fibroid diagnosis was missing for 12 participants.

[b] Data are presented as number (percentage) of participants unless otherwise indicated.

[c] Mother's educational attainment at approximately 10 years of age of the participant.

© 2024 Langton CR et al. *JAMA Network Open.*

[d] Moderate indicates 1 to 5 drinks on days when alcohol is consumed or 4 drinks or more on an occasion no more than once per month; heavy indicates 6 drinks or more on days when alcohol is consumed or 4 drinks or more on an occasion at least twice per month.

© 2024 Langton CR et al. *JAMA Network Open.*

eTable 3. Multivariable Associations of Maternal History of Fibroids With Fibroid Growth, Study of Environment, Lifestyle & Fibroids, 2010-2018

			Estimated % difference in growth (95% confidence interval)		
**Exposure**	**No. of participants**	**No. of growth intervals[a]**	**Model 1[b]**	**Model 2[c]**	**Model 3[d]**
Maternal history of fibroids					
Not diagnosed	235	735	Reference	Reference	Reference
Diagnosed	182	579	6.2 (-3.2, 16.6)	6.1 (-3.5, 16.7)	8.0 (-1.2, 18.0)
Age at maternal fibroid diagnosis[e]					
Not diagnosed	235	735	Reference	Reference	Reference
20-29 y	46	136	5.2 (-9.8, 22.8)	4.9 (-10.3, 22.7)	8.7 (-6.0, 25.8)
30-39 y	59	184	2.7 (-10.6, 17.9)	2.4 (-11.0, 17.8)	1.6 (-10.9, 15.9)
≥40 y	65	217	10.9 (-2.7, 26.3)	10.7 (-3.1, 26.3)	13.7 (0.6, 28.4)

Abbreviations: GED, General Educational Development; No., number.

[a] Growth analyses were conducted among fibroids which could be matched across successive visits based on fibroid location; this includes 417 participants with 1,314 interval growth measurements. Participants could contribute multiple fibroids and fibroids could be followed across multiple visits.

[b] Adjusted for fibroid characteristics of volume of fibroid (<0.5, 0.5-<4.2, 4.2-<14.1 vs ≥14.1 cm^3), number of fibroids (ordinal; 1, 2, 3, ≥4), and age (continuous).

[c] Adjusted for fibroid characteristics and maternal factors: educational attainment of mother at approximately 10 years of age of the participant (high school or GED or less vs some college, technical training after high school or college degree) and mother's age at enrollment of participant (continuous).

[d] Adjusted for fibroid characteristics, maternal factors, and participant factors: age at menarche (≤10, 11, 13, or ≥14 vs 12 years), and time-varying factors of years since last use of injection contraceptive (<2 vs ≥2 years including never), parity (1-2, or ≥3 vs 0 births), years since last birth (≥3 years ago including no births vs <3 years ago), current smoking (yes vs no), body mass index (calculated as measured weight in kilograms divided by measured height in meters squared) (25.0-<30.0, 30.0-<35.0, 35.0-<40.0, or ≥40.0 vs <25.0, kg/m^2), and household income (<$20,000 vs ≥$20,000).

[e] A total of 405 participants and 1,272 growth intervals were included in age at maternal fibroid diagnosis models due to mother's age at fibroid diagnosis missing for 12 participants.

© 2024 Langton CR et al. *JAMA Network Open.*

eTable 4. Sensitivity Analyses for Multivariable Association of Maternal History of Fibroids With Fibroid Incidence, Study of Environment, Lifestyle & Fibroids (SELF), 2010-2018

	**Maternal History of Fibroids**		**Age at Maternal Fibroids Diagnosis**			
		**HR (95% CI)[a]**		**HR (95% CI)[a]**		
	**N[b]**	**Diagnosed**	**N[b]**	**Age 20-29 y**	**Age 30-39 y**	**Age ≥40 y**
**Estimates from primary analyses**	**1187**	**1.21 (0.96-1.52)**	**1168**	**1.56 (1.11-2.21)**	**1.03 (0.71-1.49)**	**1.11 (0.81-1.52)**
1. Restrict to participants whose mother provided own fibroid history	1045	1.19 (0.93-1.53)	1034	1.59 (1.11-2.27)	1.03 (0.70-1.52)	1.05 (0.75-1.47)
2. Restrict to mother age ≥50 y at participant enrollment	806	1.24 (0.95-1.62)	794	1.58 (1.06-2.36)	1.08 (0.71-1.63)	1.13 (0.78-1.63)
3. Assign missing age of mother's fibroid diagnosis to age 20-29 y category	N/A	N/A	1187	1.64 (1.20-2.25)	1.02 (0.70-1.47)	1.11 (0.80-1.52)
4. Assign missing age of mother's fibroid diagnosis to age ≥40 y category	N/A	N/A	1187	1.55 (1.10-2.19)	1.02 (0.70-1.47)	1.19 (0.88-1.61)
5. Exclude participants who reported family history as reason for joining study[c]	386	1.25 (0.72-2.16)	--	--	--	--

Abbreviations: CI, confidence interval; GED, General Educational Development; HR, hazard ratio; No., number.

[a] Cox proportional hazards regression model with age as the time scale plus adjustment for maternal factors: mother's age at participant enrollment (continuous) and highest education of mother at approximately 10 years of age of the participant (high school or GED or less vs some college, technical training after high school or college degree), and participant factors: age at menarche (≤10, 11, 13, or ≥14 vs 12 years) and time-varying factors of parity (1-2, or ≥3 vs 0 births), time since last birth (≥3 years ago including no births vs <3 years ago), time since last use of injection contraceptive (<2 vs ≥2 years including never), current smoker (yes vs no), body mass index (calculated as measured weight in kilograms divided by measured height in meters squared) (25.0<30.0, 30.0-<35.0, 35.0-<40.0, or ≥40 vs <25.0 kg/m^2), and annual household income (<$20,000 vs ≥$20,000). No maternal fibroid diagnosis is reference group for all estimates.

[b] Number of participants included in analytical sample.

[c] The question that captured reasons for joining the study was not added to the pre-enrollment questionnaire until ~1 year into the study. Consequently, the analytical sample that excludes those who reported family history as a reason for joining the study is based on a reduced dataset of participants with valid responses to the reasons for joining question (n=570). Due to small numbers the sensitivity analysis was only performed for the maternal history of fibroids model.

© 2024 Langton CR et al. *JAMA Network Open.*

eTable 5. Sensitivity Analyses for Multivariable Association of Maternal History of Fibroids With Fibroid Growth, Study of Environment, Lifestyle & Fibroids (SELF), 2010-2018

	**Maternal History of Fibroids**		**Age at Maternal Fibroids Diagnosis**			
		**Estimated % difference (95% CI)[a]**		**Estimated % difference (95% CI)[a]**		
	**N[b]**	**Diagnosed**	**N[b]**	**Age 20-29 y**	**Age 30-39 y**	**Age ≥40 y**
**Estimates from primary analyses**	**417**	**8.0 (-1.2, 18.0)**	**405**	**8.7 (-6.0, 25.8)**	**1.6 (-10.9, 15.9)**	**13.7 (0.6, 28.4)**
1. Restrict to participants whose mother provided own fibroid history	374	7.3 (-2.0, 17.6)	366	8.1 (-7.0, 25.5)	0.5 (-11.9, 14.7)	11.1 (-1.7, 25.6)
2. Restrict to mother age ≥50 y at participant enrollment	336	10.6 (0.3, 22.1)	326	9.0 (-7.5, 28.3)	5.9 (-8.4, 22.5)	17.6 (2.8, 34.4)
3. Assign missing age of mother's fibroid diagnosis to age 20-29 y category	N/A	N/A	417	7.6 (-5.8, 22.8)	1.6 (-10.9, 16.0)	13.7 (0.6, 28.4)
4. Assign missing age of mother's fibroid diagnosis to age ≥40 y category	N/A	N/A	417	8.5 (-6.3, 25.6)	1.6 (-10.9, 15.9)	12.2 (0.1, 25.8)
5. Remove outliers from growth models[c]	411	7.2 (-1.6, 16.7)	400	4.2 (-9.5, 20.0)	3.0 (-9.2, 16.9)	14.2 (1.5, 28.5)
6. Exclude participants who reported family history as reason for joining study[d]	121	-0.2 (-15.9, 18.5)	--	--	--	--

Abbreviations: CI, confidence interval; GED, General Educational Development; HR, hazard ratio; No., number.

[a] Adjusted for age (continuous), fibroid characteristics: volume of fibroid (<0.5, 0.5-<4.2, or 4.2-<14.1 vs ≥14.1 cm^3), number of fibroids (ordinal; 1, 2, 3, or ≥4), maternal factors: education attainment of mother at approximately 10 years of age of the participant (high school or GED or less vs some college, technical training after high school or college degree) and mother's age at enrollment of participant (continuous) and participant factors: age at menarche (ordinal: ≤10, 11, 13, or ≥14 vs 12 years), and time-varying factors of years since last use of injection contraceptive (<2 vs ≥2 years including never), parity (1-2, or ≥3 vs 0 births), years since last birth (≥3 years ago including no births vs <3 years ago), current smoking (yes vs no), body mass index (calculated as measured weight in kilograms divided by measured height in meters squared) (25.0-<30.0, 30.0-<35.0, 35.0-<40.0, or ≥40 vs <25.0 kg/m^2), and annual household income (<$20,000 vs ≥$20,000). No maternal fibroid diagnosis is reference group for all estimates.

[b] Number of participants included in analytical sample.

[c] Uterine fibroids with residuals for growth >3 standard deviations from the mean were removed from the growth models.

© 2024 Langton CR et al. *JAMA Network Open.*

[d] The question that captured reasons for joining the study was not added to the pre-enrollment questionnaire until ~1 year into the study. Consequently, the analytical sample that excludes those who reported family history as a reason for joining the study is based on a reduced dataset of participants with valid responses to the reasons for joining question (n=182). Due to small numbers the sensitivity analysis was only performed for the maternal history of fibroids model.

© 2024 Langton CR et al. *JAMA Network Open.*
