# Supplementary material for: Family History and Uterine Fibroid Development in Black and African American Women
Source: JAMA Netw Open. 2024 Apr 3;7(4):e244185. doi: 10.1001/jamanetworkopen.2024.4185 (PMC10993075; doi:10.1001/jamanetworkopen.2024.4185)
Supplement: Supplement 2. — Data Sharing Statement [file jamanetwopen-e244185-s002.pdf]

# Data Sharing Statement

Langton. Family History and Uterine Fibroid Development in Black and African American Women. *JAMA Netw Open*. Published April 03, 2024.
doi:10.1001/jamanetworkopen.2024.4185

**Data**
**Data available:** Yes
**Data types:** Deidentified participant data
**How to access data:** Deidentified participant data that underlie the primary results reported in this article can be requested for purposes of replication or meta-analysis by emailing baird@niehs.nih.gov and quaker.harmon@nih.gov. All data releases will require a data use proposal and a data use agreement and will comply with the IRB and consent of the SELF study, which may require omission of some data elements. The NIH IRB may be asked to review the request and study consent forms to approve a data transfer.
**When available:** With publication

**Supporting Documents**
**Document types:** None

**Additional Information**
**Who can access the data:** Researchers whose proposed use of the data has been approved.
**Types of analyses:** For purposes of replication or meta-analysis.
**Mechanisms of data availability:** All data releases will require a data use proposal and a data use agreement and will comply with the IRB and consent of the SELF study, which may require omission of some data elements. The NIH IRB may be asked to review the request and study consent forms to approve a data transfer.
